# Expression of tumor-associated carbohydrate antigen Tn in cancer cells is stimulated by cytokine secreted from cancer cells

**DOI:** 10.1186/2051-1426-2-S3-P170

**Published:** 2014-11-06

**Authors:** Alex Lin, Chiao-en Chen, Jaulang Hwang

**Affiliations:** 1 Dept. Biochemistry, School of Medicine, Taipei Medical University, Taipei, Taiwan

## 

Tumour-associated carbohydrate antigen (TACA) Tn is always observed on the cell surface of malignant tumour cells. Tn is presented as mucin-type carbohydrate. It has been reported to be associated with cancer progression, but the regulation on Tn expression in cancer cells is not fully understood. In this study, we took an effort to explore the mechanism of how Tn expression is regulated in cancer cells. We observed that Tn expression in cancer cells was decreased by changing to fresh medium every 24 h. If we keep cancer cells in continuous cultured medium, we observed that Tn expression was elevated. We further showed that adding continuous cultured medium to cancer cells which change to fresh medium every 24 hours resulted in an induction of Tn expression. These observations suggest that cancer cells may autocrine some substance to stimulate Tn expression. We have identified at least two cytokines, TNF-α and IL-6, that are autocrined by cancer cells and both cytokines can induce Tn expression. We further demonstrated that the induction of Tn expression can be driven by ras oncogene. This study thus indicates that tumor microenviroment contributed by cancer cell oncogene and by autocrine of cytokines is an important factor in regulation of Tn expression.

